# Inhibition of Fas associated phosphatase 1 (Fap1) facilitates apoptosis of colon cancer stem cells and enhances the effects of oxaliplatin

**DOI:** 10.18632/oncotarget.25401

**Published:** 2018-05-25

**Authors:** Weiqi Huang, Ling Bei, Elizabeth A. Eklund

**Affiliations:** ^1^ Feinberg School of Medicine, Northwestern University, Chicago IL, USA; ^2^ Jesse Brown Veteran's Administration Medical Center, Chicago IL, USA

**Keywords:** stem cell, phosphatase, Fas, apoptosis, gene transcription

## Abstract

Fas associated phosphatase 1 (Fap1) is a ubiquitously expressed protein tyrosine phosphatase. Fap1 substrates include Fas and Gsk3β, suggesting a role in regulating cell survival. Consistent with this, increased Fap1 expression is associated with resistance to Fas or platinum induced apoptosis in some human colon cancer tumors or cell lines. In the current studies, we found that Fap1 expression was significantly greater in CD133^+^ colon cancer stem cells compared to CD133^−^ tumor cells. *PTPN13* promoter activity (encoding Fap1) was repressed by interferon regulatory factor 2 (irf2), and expression of Fap1 and Irf2 were inversely correlated in CD133^+^ or CD133^−^ colon cancer cells. We determined that CD133^+^ cells were relatively resistant to Fas or oxaliplatin induced apoptosis, but this was reversed by Fap1-knockdown or a Fap1-blocking tripeptide (SLV). In a murine xenograft model of colon cancer, we found treatment with SLV peptide significantly decreased tumor growth and relative abundance of CD133^+^CD44^+^ cells; associated with increased phosphorylation of Fap1 substrates. SLV peptide also enhanced inhibitory effects of oxaliplatin on tumor growth and Fap1 substrate phosphorylation in this model. Our studies suggest that therapeutically targeting Fap1 may decrease persistence of colon cancer stem cells during treatment with platinum chemotherapy by activating Fap1 substrates. In a murine model of chronic myeloid leukemia, we previously determined that inhibition of Fap1 decreased persistence of leukemia stem cells during tyrosine kinase inhibitor treatment. Therefore, Fap1 may be a tissue agnostic target to increase apoptosis in malignant stem cells.

## INTRODUCTION

Approximately 4% of individuals in the USA will be diagnosed with colorectal cancer (CRC) during their lifetime [1]. Once distant metastases develop, no current therapeutic approaches prolong survival in this disease [2]. Tumors that recur after chemotherapy or at metastatic sites are less sensitive to platinum-based chemotherapy compared to primary colon cancer tumors [3]. The mechanism for this is not known, but such tumors are hypothesized to be relatively enriched for colon cancer stem cells (CSC) compared to primary and/or chemotherapy naïve tumors [4]. Treatment approaches specifically targeting CSCs may be beneficial for decreasing tumor recurrence or metastatic potential.

Colon cancer stem cells are identified by expression of CD133, CD44, CD26 and Lgr5, although some data suggests this profile may be modulated during metastasis, *ex vivo* cell manipulation, or passage in culture [5–11]. Relative quiescence of these cells is hypothesized to render them less sensitive to cell cycle-active chemotherapeutic agents such as cis-platinum or oxaliplatin [5]. Malignant stem cells are also hypothesized to be relatively Fas resistant. In the current studies, we hypothesize that Fas-resistance of some colon cancer stem cells is due to increased expression of Fap1; a ubiquitously expressed protein tyrosine phosphatase [12]. Fap1 expression is increased in metastatic versus primary tumors, with increasing Duke's stage, and after treatment with platinum versus in chemotherapy naive tumors [13]. However, relative Fap1 expression in various tumor cell populations has not been investigated.

Fap1 substrates include Fas and Gsk3β [14, 15]. Fap1 interacts with the Fas C-terminus through a Fap1-PDZ domain; dephosphorylating Fas and inhibiting apoptosis [14]. Other investigators identified an inverse correlation between Fap1 and Fas-induced apoptosis in some colon cancer cell lines, or platinum induced apoptosis in some primary patient CRC samples [14, 16, 17]. A tripeptide representing the Fas C-terminus (SLV) blocks the Fap1-PDZ domain and prevents interaction of Fap1 with partner proteins [18, 19]. Consistent with this, SLV peptide restored Fas-induced apoptosis in colon cancer cell lines with increased Fap1, and cisplatin sensitivity in samples from patients with platinum-insensitive tumors [14].

We determined that interaction of Fap1 with Apc (the adenomatous polyposis coli protein) results in dephosphorylation (inactivation) of Gsk3β by Fap1 [19]. Since phosphorylation of βcatenin by Gsk3β results in βcatenin ubiquitination and proteasomal degradation, Fap1 stabilizes βcatenin through this mechanism [15]. We found that SLV peptide blocked Fas-resistance and βcatenin-activation in Fap1 overexpressing leukemia cells [15, 20]. Fap1 expression is increased in CD34^+^ leukemia stem cells (LSCs) from chronic myeloid leukemia (CML) patients and further increases upon disease progression [12]. We also found that Fap1 contributed to persistence of CML-LSCs during tyrosine kinase inhibitor treatment; facilitating relapse [20].

We determined that transcription of the *PTPN13* promoter (encoding Fap1) was repressed by Icsbp/Irf8 (interferon consensus sequence binding protein/interferon regulatory factor 8) in myeloid leukemia cells [21]. Although expression of Icsbp is myeloid restricted, other interferon regulatory factors are expressed in colon cancer cells. Specifically, Irf2 is expressed in CRC cells and polymorphisms in the *IRF2* gene are implicated in the pathogenesis of this disease [22].

In the current studies, we investigate the impact of Fap1 on tumor growth in a murine xenograft model of colon cancer. We also study regulation of Fap1 expression and the relative influence of Fap1 on CRC-CSCs versus other cell populations in the tumors. Based on these results, we hypothesize Fap1 influences the biology of malignant stem cells in a tissue agnostic manner in neoplasms as diverse as CRC and CML, and might be a rationale therapeutic target to prevent relapse, and/or effect cure, in a number of cancers.

## RESULTS

### Fap1 is increased in CD133^+^ colon cancer cells

**Figure 1:**
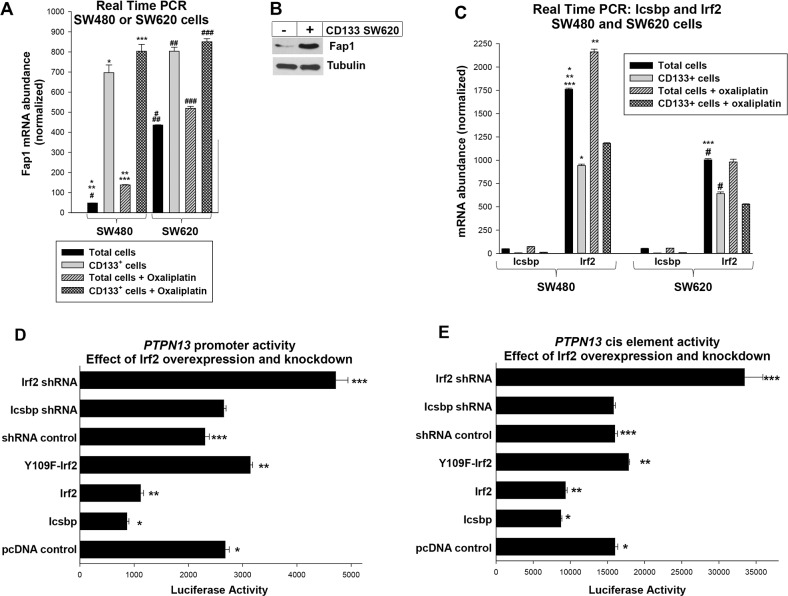
Fap1 expression is increased in CD133^+^ colon cancer cells and the *PTPN13* promoter is regulated by Irf2 **(A)** Expression of Fap1 is correlated with CD133^+^ cell abundance in colon cancer cell lines. Total SW480 or SW620 cells, or CD133^+^ cells isolated from these lines, were analyzed for Fap1 mRNA by real time PCR. Significant differences are indicated by ^*^, ^**^, ^***^, ^#^, ^##^, or ^###^ (p<0.01, n=6 for all comparisons). **(B)** Fap1 protein is more abundant in CD133^+^ cells vs CD133^−^ cells. CD133^+^ cells that were isolated from the SW620 line were compared to CD133^−^ cells by Western blot of total lysate proteins. Blots were probed from Fap1 and Tubulin (as a loading control). **(C)** Irf2 expression is decreased in CD133^+^ cells vs the total SW480 or SW620 cell population, and significantly less Icsbp is expressed in these cells compared to Irf2. Total SW480 or SW620 cells, or CD133^+^ cells isolated from these lines, were analyzed for Icsbp or Irf2 mRNA by real time PCR. Significant differences are indicated by ^*^, ^**^, ^***^, or ^#^ (p<0.01, n=6 for all comparisons). **(D)** Irf2 represses the *PTPN13* promoter. SW620 cells were transfected with a construct with 1.0 kb of *PTPN13* promoter linked to a luciferase reporter and vectors to overexpress Icsbp, wild type Irf2, or Y109F-Irf2 (versus empty control vector), or to knockdown Icsbp or Irf2 (with specific shRNA versus scrambled shRNA vector). Significant differences indicated by ^*^, ^**^, or ^***^ (p<0.001, n=6 for all comparisons). **(E)** Irf2 represses the Icsbp-binding cis element from the *PTPN13* promoter. SW620 cells were transfected with a construct with three copies of the previously identified *PTPN13* cis element linked to a minimal promoter and luciferase reporter. Cells were co-transfected with vectors to overexpress or knockdown Icsbp or Irf2, as described above. Significant differences indicated by ^*^, ^**^, or ^***^ (p<0.001, n=6 for all comparisons).

Fap1 expression inversely correlates with sensitivity to Fas-induced apoptosis in some colon cancer cell lines [23]. This includes SW480; a Fas sensitivity line with relatively low Fap1 expression that was derived from a primary colon cancer tumor [23, 24]. SW620 was derived from a metastatic lesion from the same patient, but has not been directly compared to SW480 cells for Fap1 expression or Fas-sensitivity. We found significantly more Fap1 in SW620 versus SW480 cells, consistent with increased Fap1 expression upon disease progression (Figure 1A) [13]. We performed additional studies to determine the mechanism for this difference between primary and metastatic CRC tumors.

We first investigated the possibility that increased Fap1 was a characteristic of cancer stem cells in particular, rather than metastatic tumors in general, by determining Fap1 expression in CD133^+^ populations from these lines. We found significantly more Fap1 mRNA in CD133^+^ cells from either line compared to the total cell population (p<0.001, n=6) (Figure 1A). Consistent with this, Fap1 protein was more abundant in CD133-selected cells versus the total population (Figure 1B). Importantly, Fap1-expression was not significantly different in CD133^+^ SW480 cells versus CD133^+^ SW620 cells (p=0.07, n=6) (Figure 1A).

Since Fap1 inversely correlates with platinum sensitivity, we determined the effect of oxaliplatin on Fap1 expression. In assays with total cell populations, oxaliplatin significantly increased Fap1 mRNA in both lines, but expression was still significantly greater in SW620 versus SW480 cells (p<0.001, n=6) (Figure 1A). However, oxaliplatin did not significantly increase Fap1 expression in the CD133^+^ population from SW480 or SW620 cells (p=0.05, n=6) (Figure 1A). Therefore, differences in Fap1 expression in metastatic lesions versus primary CRC tumors, or pre versus post oxaliplatin, were explained by the relative abundance of CD133^+^ cells.

### Irf2 regulated PTPN13 promoter activity in colon cancer cells

We were interested transcriptional regulation of the gene encoding Fap1 (*PTPN13*) in CRC cells. However, Icsbp/Irf8-expression, which regulates this gene in hematopoietic cells, is generally restricted to differentiating phagocytes or B cells [25]. Since this Icsbp/Irf8 protein shares some target genes with the ubiquitously expressed Irf2 (interferon regulatory factor 2), we investigated relative expression of the two in the CRC cell lines [26].

Irf2 was easily detected in SW480 or SW620 cells, but was significantly less abundant in the latter (p<0.01, n=6 comparing the lines) (Figure 1C). For both lines, Irf2 mRNA abundance was significantly less in CD133^+^ cells versus the total population (p<0.01, n=6) (Figure 1C). Therefore, expression of Irf2 inversely correlated with Fap1. Expression of Icsbp/Irf8 was more than a log less than Irf2 in both lines and in CD133^+^ cells (Figure 1C).

To investigate *PTPN13* promoter activity, we transfected SW620 cells with a construct containing 1.0 kb of 5′ flank linked to a luciferase reporter (or empty reporter vector) [21]. Cells were co-transfected with vectors to overexpress Icsbp, Irf2, Y109F-Irf2, or control vectors; or shRNAs specific to Icsbp or Irf2, or scrambled shRNA control vectors. We found that overexpression of either Icsbp or wild type Irf2 decreased *PTPN13* promoter activity (p<0.0001, n=4), but Y109F-Irf2 did not (Figure 1D). Phosphorylation of Y109 is required for Irf2-binding to some target genes [21]. And, knockdown of Irf2 significantly increased *PTPN13*/reporter construct activity (versus scrambled control) (p<0.001, n=4), but knockdown of Icsbp/Irf8 did not (consistent with low levels of expression of endogenous Icsbp in these cells, p=0.7, n=4) (Figure 1D). Neither overexpression nor knockdown of either protein influenced expression of control, empty reporter vector in these studies (subtracted as background). Results were normalized for transfection efficiency using a constitutively active renilla luciferase control vector.

We performed additional studies to determine if Irf2 and Icsbp influenced the same *PTPN13* cis element [21]. For these studies, SW620 cells were transfected with a luciferase reporter vector with three copies of the Icsbp-binding site from the *PTPN13* promoter linked to a minimal promoter (or empty minimal promoter/reporter control vector). Cells were co-transfected with vectors to overexpress or knockdown Icsbp and Irf2 (or relevant control vectors), as described above. We found the *PTPN13* cis element was equivalently repressed by overexpressed Icsbp or Irf2, but only activated by knockdown of Irf2 (Figure 1E). Neither knockdown nor overexpression of these proteins influenced activity of the empty, minimal promoter/reporter vector (subtracted as background). Cells were co-transfected with a constitutively active renilla luciferase reporter vector as an internal control for transfection efficiency.

### The SW620 cell line is relatively enriched for CD133^+^ cells compared to SW480 cells

**Figure 2:**
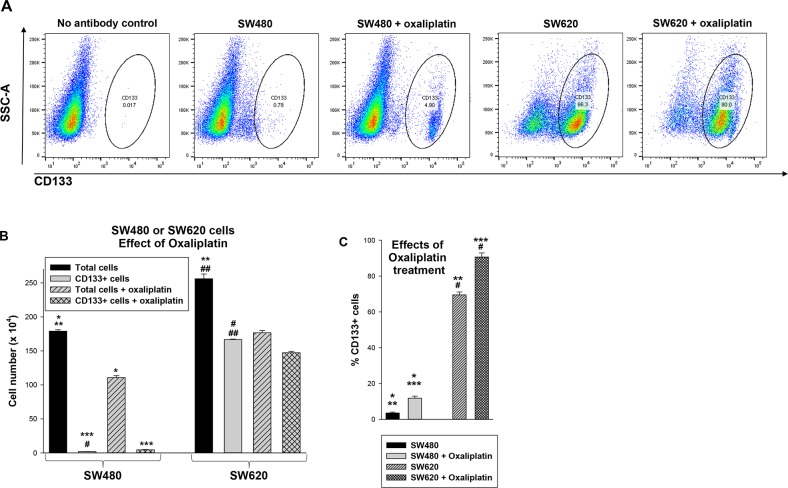
Treatment with oxaliplatin decreases total SW480 or SW620 cells, but increases the relative number of CD133^+^ cells in these lines **(A)** SW480 and SW620 cells were analyzed by flow cytometry for CD133^+^ cells with versus without oxaliplatin treatment. Histograms were gated using unstained cells as a control. **(B)** Oxaliplatin treatment decreased the total number of SW480 or SW620 cells, but the number of CD133^+^ cells were relatively unaffected. Absolute numbers of cells under the various conditions were determined. Statistically significant differences are indicated by ^*^, ^**^, ^***^, ^#^, or ^##^ (p<0.0001, n=6 for all comparisons). **(C)** The percent of CD133^+^ cells was determined before or after oxaliplatin treatment in both lines. Significant differences indicated by ^*^, ^**^, ^***^, or ^#^ (p<0.001, n=6 for all comparisons).

Results of studies using total vs CD133^+^ cells from the SW480 and SW620 lines suggested a difference in relative abundance of colon cancer stem cells in the two lines. Specifically, these results suggested CD133^+^ cells were relatively more abundant in the SW620 metastatic line compared to the SW480 primary tumor line. As an initial approach to this question, we performed flow cytometry for CD133^+^ cells in the two lines (Figure 2A). Consistent with our hypothesis, we found an increase in the absolute number (Figure 2B) (p<0.001, n=6) and relative percent (Figure 2C) (p<0.001, n=6) of CD133^+^ cells in SW620 versus SW480 cells.

Since CSCs are hypothesized to be relatively chemotherapy resistant, we examined the effect of oxaliplatin on the total cell population and CD133^+^ subset in both lines. We found that oxaliplatin decreased the total number of SW480 or SW620 cells (p<0.0001, n=6) (Figure 2B), but increased the relative abundance of CD133^+^ cells (p<0.001, n=6) (Figure 2C). This effect was greater in SW480 versus SW620 cells (~66% increase versus ~20% increase, p<0.01, n=6) (Figure 1C). This was consistent with a greater sensitivity of CD133^−^ cells to oxaliplatin compared CD133^+^ cells, and a greater abundance of CD133^−^ cells in the SW480 line.

### Fap1 contributed to Fas-resistance in colon cancer stem cells

**Figure 3:**
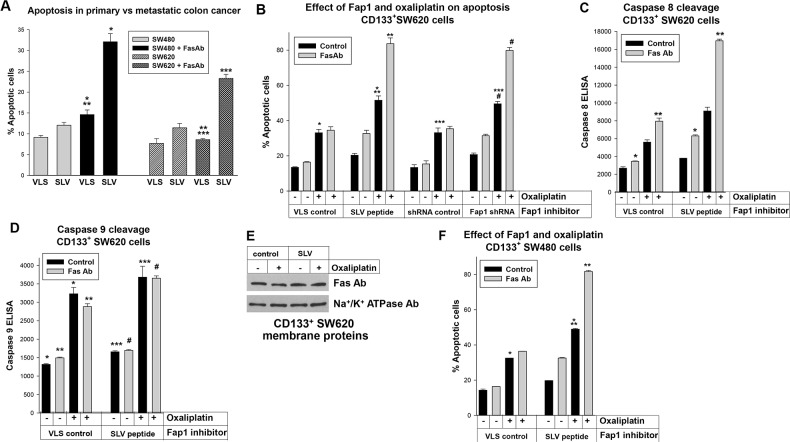
Fap1-inhibition increased Fas or oxaliplatin induced apoptosis in CD133^+^ colon cancer cells **(A)** Fap1-blocking SLV peptide increases Fas-induced apoptosis. Total SW480 or SW620 cells were treated with SLV peptide (or VLS control), with or without Fas-agonist antibody, and analyzed for apoptosis by Annexin V staining. Significant differences indicated by ^*^, ^**^, or ^***^ (p<0.01, n=4 for all comparisons). **(B)** Fap1-inhibition increases Fas or oxaliplatin induced apoptosis in CD133^+^ SW620 cells. CD133^+^ SW620 cells were analyzed for apoptosis by Annexin V staining, with or without Fas-agonist antibody. Some cells were treated with SLV peptide (or VLS control) or transduced with a vector to express Fap1 specific shRNAs (or scrambled control). Significant differences indicated by ^*^, ^**^, ^***^, or ^#^ (p<0.001, n=4 for all comparisons). **(C)** Fap1-inhibition increases Fas induced caspase 8 cleavage in CD133^+^ SW620 cells. Cells were analyzed by ELISA for cleavage of caspase 8 (death receptor induced apoptosis). Statistically significant differences indicated by ^*^ or ^**^ (p<0.001, n=4). **(D)** Oxaliplatin treatment increases caspase 9 cleavage in CD133^+^ SW620 cells. Cells were analyzed by ELISA for cleavage of caspase 9 (intrinsic apoptosis). Statistically significant differences indicated by ^*^, ^**^, ^***^, or ^#^ (p<0.001, n=4). **(E)** Plasma membrane expression of Fas was not altered by SLV peptide or oxaliplatin treatment of CD133^+^ SW620 cells. Western blots of cell lysates were probed for Fas or Na+/K+ ATPase (as a loading control). **(F)** Fap1 inhibition increases Fas or oxaliplatin induced apoptosis in CD133^+^ SW480 cells. Similar experiments were performed with the SW480 cell line. Statistically significant differences are indicated by ^*^ or ^**^ (p<0.001, n=6 for both comparisons).

We next investigated the influence of Fap1 on Fas or platinum induced cell death in CD133^+^ CRC cells. In the first set of experiments, apoptosis was determined by flow cytometry for Annexin V staining with versus without a Fas agonist antibody (4 independent experiments, assayed in triplicate). We found SW480 cells were significantly more sensitive to Fas-induced apoptosis than SW620 cells (p<0.001, n=4) (Figure 3A). Treatment with Fap1-blocking, SLV peptide increased the apoptotic response to the Fas agonist antibody in SW480 cells (62.5% ± 1.2% increase in apoptosis with Fas antibody in SLV treated cells versus a 37.6% ± 0.8% increase with Fas antibody in VLS treated control cells; p<0.01, n=4) and SW620 cells (51.1% ± 1.5% increase in apoptosis with Fas antibody in SLV treated cells versus 10.0% ± 0.3% increase with Fas antibody in VLS treated control cells; p<0.01, n=4).

Therefore, Fap1 inhibition had a relatively greater effect on Fas resistance in the metastatic, SW620 line. Since these differences might be due to CD133^+^ cell abundance in SW620 versus SW480 lines, we investigated the impact of Fap1 on Fas-resistance in these cells. CD133^+^ SW620 or SW480 cells were isolated and analyzed for apoptosis with or without Fas agonist antibody, as above. Cells were treated with SLV or VLS control peptide, or transduced with vectors to express Fap1-specific shRNAs (or scrambled control).

We found CD133^+^ SW620 cells were resistant to Fas-induced apoptosis, but either SLV peptide or Fap1-knockdown significantly increased Fas sensitivity (p<0.001, n=4 comparing % apoptotic cells after Fas antibody ± Fap1 inhibition) (Figure 3B). Oxaliplatin significantly increased baseline apoptosis in CD133^+^ SW620 cells (p<0.001, n=4), but did not increase the sensitivity of these cells to Fas-agonist antibody (Figure 3B). However, the combination of oxaliplatin plus SLV peptide or Fap1 knockdown significantly increased Fas-induced apoptosis (p<0.001, n=4 comparing the Fas-antibody induced apoptosis in oxaliplatin treated cells ± Fap1-inhibition) (Figure 3B). Therefore, Fap1 inhibition increased sensitivity to Fas and oxaliplatin in CD133^+^ SW620 cells.

Oxaliplatin increased intrinsic apoptosis in the absence of Fap1 inhibition or Fas-induced apoptosis. Therefore, we also assayed for extrinsic apoptosis versus intrinsic apoptosis by determining cleavage of caspase 8 versus 9, respectively (by ELISA). We found that treatment with SLV peptide resulted in a significant increase in Fas-antibody induced caspase 8 cleavage in CD133^+^ SW620 cells (p<0.0001, n=4) (Figure 3C). The addition of oxaliplatin to SLV peptide significantly increased the percent of Fas-antibody induced caspase 8 cleavage in these cells (p<0.001, n=4). In contrast, oxaliplatin significantly increased caspase 9 cleavage in these cells (p<0.001, n=4), but SLV peptide did not (p=0.2, n=4) (Figure 3D). We also investigated the effect of oxaliplatin and Fap1-inhibition on Fas expression in control experiments. For these studies, plasma membrane proteins were isolated from CD133^+^ SW620 cells and analyzed by Western blot (with Na+/K+ ATPase as a loading control). Neither treatment altered Fas expression in these cells (Figure 3E).

We performed similar studies in CD133^+^ SW480 cells. We found resistance to Fas and oxaliplatin induced apoptosis was equivalent in CD133^+^ SW480 and CD133^+^ SW620 cells (p>0.1, n=4) (Figure 3F). We also found an equivalent impact of Fap1-inhibition on CD133^+^ cells from the two lines (Figure 3F). These studies suggested CD133^+^ colon cancer cells from primary or metastatic tumors were equivalently resistant to oxaliplatin or Fas induced apoptosis, but this was reversed by inhibition of Fap1.

### Fap1 inhibition decreased growth of colon cancer xenografts and delayed relapse after oxaliplatin treatment

To investigate the impact of Fap1 on tumor growth and oxaliplatin sensitivity *in vivo*, we used a murine xenograft model. For these studies, SW620 cells were injected in the flanks of athymic *Nude* mice. Once tumors were >200 mm^3^, mice were treated by daily intraperitoneal (IP) injection of Fap1-blocking SLV peptide or VLS control peptide (12 per group). Tumors were measured twice weekly and mice with tumors >2,000 mm^3^ were sacrificed.

**Figure 4:**
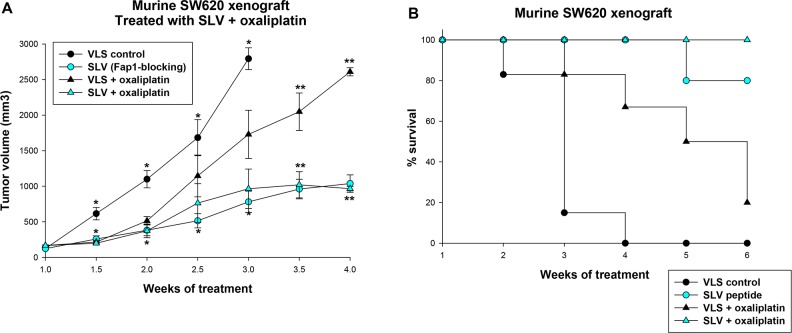
Fap1-inhibition decreases tumor growth in a murine xenograft model of colon cancer SW620 cells were injected in the flanks of athymic Nude mice and tumor volume was determined biweekly. Some mice were injected daily with Fap1-blocking SLV peptide and others with VLS control peptide (n=12 per cohort). Other mice were treated weekly with oxaliplatin (day 0, 7 and 14) and daily with Fap1 blocking SLV peptide or VLS control peptide (n=12 per cohort). **(A)** Treatment with SLV peptide decreases the rate of tumor growth with or without oxaliplatin. Significant differences in tumor size at the same time point are indicated by ^*^ or ^**^ (p<0.01, n=12). **(B)** Treatment with oxaliplatin or SLV peptide increases survival, and the combination increases survival more than either alone. The percent of surviving mice during SLV versus VLS peptide treatment over time was determined (Χ^2^=5.52, p=0.02, n=12 for comparison of mice treated with VLS vs SLV peptide, and Χ^2^=6.2, p=0.01, n=12 for comparison of mice treated with oxaliplatin with VLS versus SLV peptide).

Tumors grew significantly more slowly in mice treated with SLV peptide compared to VLS control (Figure 4A). Indeed, a significant difference in tumor size was detected by 1.5 weeks of treatment which persisted throughout the experiment (p<0.001, n=12). In the VLS control group, no mice survived the 3^rd^ week of the experiment, but 80% of SLV peptide treated mice survived 6 weeks, representing a statistically significant survival improvement (p<0.001, n=12) (Figure 4B).

Since oxaliplatin is the cornerstone of contemporary CRC treatment [26], we also examined the impact of Fap1-inhibition on relapse after treatment with this agent. For these studies, athymic *Nude* mice were injected with SW620 cells and treatment with SLV peptide or VLS control was initiated, as above. Cohorts were simultaneously treated with oxaliplatin (once a week for three weeks; 12 per group). Treatment with SLV or VLS peptide was continued until mice were sacrificed, according to the parameters described above.

We found that SLV peptide treatment significantly delayed tumor growth after oxaliplatin treatment in comparison to treatment with VLS control peptide (p<0.001, n=12 for later time points) (Figure 4A). Survival was significantly prolonged by oxaliplatin treatment compared to treatment with VLS control peptide alone and was prolonged in oxaliplatin treated mice by the addition of SLV peptide (p<0.001, n=12) (Figure 4B).

**Figure 5:**
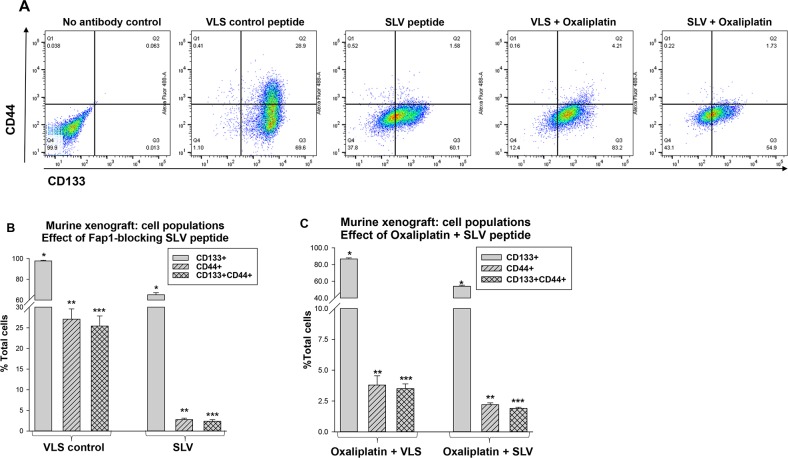
Fap1-inhibition decreases abundance of CD133^+^CD44^+^ cells in in a murine xenograft model of colon cancer with or without oxaliplatin Tumors from the mice described above were analyzed for cell population distribution after various treatments. **(A)** Histograms from flow cytometry demonstrate decreased abundance of CD133^+^CD44^+^ cells after treatment with SLV peptide with or without oxaliplatin. A representative histograms for each cohort is shown. **(B)** Treatment with SLV peptide decreases relative abundance of CD133^+^CD44^+^ cells in xenograft tumors. Tumors were simultaneously harvested from mice treated with SLV peptide versus VLS control (when control group tumors were >2,000 mm^3^) and analyzed for CD133 and CD44 expression by flow cytometry. Significant differences indicated by ^*^, ^**^, or ^***^. (p<0.001, n=6). **(C)** The addition of SLV peptide to oxaliplatin treatment decreases relative abundance of CD133^+^CD44^+^ cells in xenograft tumors. Tumors were simultaneously harvested from mice treated with oxaliplatin + SLV peptide versus VLS control (when control group tumors were >2,000 mm^3^) and analyzed for CD133 and CD44 expression by flow cytometry. Significant differences indicated by ^*^, ^**^, or ^***^. (p<0.001, n=6).

We examined tumors harvested simultaneously from the treatment groups at various time points (i.e. tumors were obtained from an SLV peptide treated mouse when VLS control treated mice were sacrificed due to tumor size). Disaggregated cells were analyzed by flow cytometry for populations associated with CRC-CSC activity; CD133^+^CD44^+^ (Figure 5A). These cells represented 25.9% ± 2.0% of total cells in tumor from VLS treated mice, but only 2.4% ± 0.2% of total cells in tumors from SLV treated mice (p<0.001, n=6) (Figure 5B). Total CD133^+^ cells in tumors from VLS versus SLV treated mice were 98.2% ± 2.0% versus 64.2% ± 2.1%, respectively (p<0.001, n=6).

We also found the addition of SLV peptide to oxaliplatin significantly decreased relative abundance of both CD133^+^ cells (54.1% ± 1.2% versus 86.7% ± 1.3% with versus without SLV) and CD133^+^CD44^+^ cells (1.7% ± 0.2% versus 3.5% ± 0.4% with versus without SLV) (p<0.001, n=6) (Figure 5C). Relative abundance of CD133^+^ or CD133^+^CD44^+^ cells was significantly less in tumors from oxaliplatin treated mice compared to mice treated with VLS or SLV peptide alone (p<0.02, n=6 for oxaliplatin + VLS versus VLS or SLV alone).

### Fap1-inhibition increased phosphorylation of Fas and Gsk3β in colon cancer xenografts

**Figure 6:**
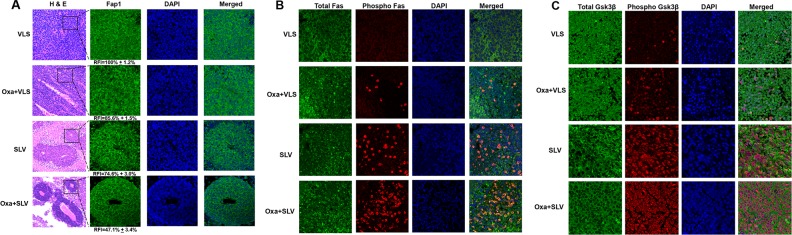
Fap1-inhibition with SLV peptide increases phosphorylation of Fap1-substrates Fas and Gsk3β in a murine xenograft model SW620 cells were injected in the flanks of athymic Nude mice and tumor volume was determination biweekly. Mice were treated weekly with oxaliplatin (days 0, 7 and 14) and injected daily with Fap1 blocking SLV peptide or VLS control peptide, or treated with SLV or VLS peptide alone (n=12 per cohort). Tumors were simultaneously harvested from cohorts of mice when control tumors were >2,000 mm3. **(A)** SLV peptide increases gland formation in xenograft tumors with or without oxaliplatin. Histology was analyzed by hematoxylin/ eosin staining. Fap1 expression was determined by immunofluorescence. Relative fluorescent intensity (RFI) of Fap1 staining is indicated below relevant panels. **(B)** SLV peptide increases Fas-phosphorylation in xenograft tumors with or without by oxaliplatin. Immunofluorescent detection of total versus phospho-Fas was performed with DAPI staining of nuclei. Areas without gland formation were selected for this study. **(C)** SLV peptide increases Gsk3β-phosphorylation with or without oxaliplatin. Immunofluorescent detection of total versus phospho- Gsk3β was performed with DAPI staining of nuclei. Areas without gland formation were selected for this study.

We were interested in determining the impact of Fap1-inhibition on phosphorylation of Fap1 substrates in the xenograft tumors. We initially examined the histology of tumors from mice treated with VLS, SLV, oxaliplatin + VLS, or oxaliplatin + SLV (Figure 6A). We found tumors from mice treated with oxaliplatin + VLS or VLS alone were highly disorganized (Figure 6A). However, tumors from mice treated with SLV peptide, with or without oxaliplatin, exhibited some areas of gland formation. This was consistent with the decrease in CD133^+^ cells in tumors from mice treated with SLV peptide, with or without oxaliplatin. We also examined these tumors for Fap1 expression (by immunofluorescent microscopy). We found that fluorescent intensity of Fap1 antibody stained cells was significantly decreased by treatment with oxaliplatin or SLV peptide (p<0.001, n=6), but further decreased by the combination of the two (p<0.01, n=6) (Figure 6A).

We also performed immunohistochemistry to determine expression and tyrosine phosphorylation state of Fap1 substrates. We found treatment with SLV peptide significantly increased Fas phosphorylation, but not total Fas protein (Figure 6B). This effect was further enhanced by combined treatment with SLV peptide and oxaliplatin. We found similar results in studies of phospho-Gsk3β (Figure 6C). Therefore, increased cell death and decreased tumor growth in mice treated with Fap1-inhibitor correlated with increased phosphorylation of Fap1 substrates *in vivo*.

**Figure 7:**
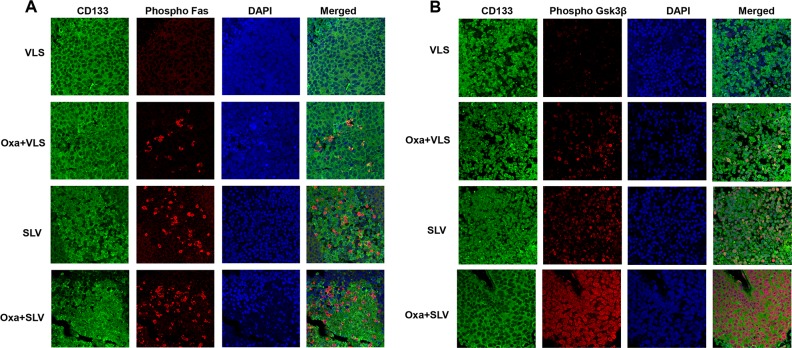
Fap1-inhibition with SLV peptide increases Fas and Gsk3β phosphorylation in CD133^+^ cells in a murine xenograft model SW620 cells were injected in the flanks of athymic Nude mice and tumor volume was determination biweekly. Mice were treated weekly with oxaliplatin (days 0, 7 and 14) and injected daily with Fap1 blocking SLV peptide or VLS control peptide, or treated with SLV or VLS peptide alone (n=12 per cohort). Tumors were simultaneously harvested from cohorts of mice when control tumors were >2,000 mm3. **(A)** SLV peptide increases Fas phosphorylation in CD133^+^ xenograft tumors with or without oxaliplatin. Immunofluorescent detection of phospho-Fas or CD133 was performed with DAPI staining of nuclei. **(B)** SLV peptide increases Gsk3β phosphorylation in CD133^+^ xenograft tumors with or without oxaliplatin. Immunofluorescent detection of phospho-Gsk3β or CD133 was performed with DAPI staining of nuclei.

We were interested in determining if Fap1-inhibition activated Fas or Gsk3β in CD133^+^ colon cancer stem cells. To investigate this, xenograft tumors from mice treated with VLS control peptide, SLV Fap1-blocking peptide, or oxaliplatin with VLS or SLV peptide were analyzed for co-localization of CD133 and phospho-Fas or phospho-Gsk3β. We found phosphorylation of Fas (Figure 7A) or Gsk3β (Figure 7B) in viable CD133^+^ tumor cells from mice treated with SLV peptide, which increased in mice undergoing combined treatment with oxaliplatin and SLV.

## DISCUSSION

Our findings suggest Fap1 expression is a characteristic of CD133^+^ colon cancer cell stem cells. Our studies also suggest that increased Fap1 expression in metastasis versus primary tumors, or pre versus post platinum chemotherapy, is related to relative abundance of these cells in the tumor. In SW480/SW620 CD133^+^ colon cancer cells, we identified an association between increased Fap1 and decreased sensitivity to either Fas or oxaliplatin induced apoptosis. And, Fap1 inhibition increased Fas-induced apoptosis and re-sensitized these cells to oxaliplatin *in vitro*. In a murine xenograft model, Fap1 inhibition was associated with decreased tumor growth, delayed progression after oxaliplatin, decreased relative abundance of CD133^+^CD44^+^ cells, and phosphorylation of Fap1 substrates (Fas and Gsk3β).

These studies also suggest Fap1 or related substrates might be rationale therapeutic targets to decrease metastasis and delay disease progression in colon cancer. Cancer stem cell-targeting is essential to cure any malignancy, and Fap1 is a potential target in these cells. Although prior studies of Fap1 investigated effects on cisplatin responsiveness *in vitro*, we chose to study oxaliplatin *in vivo* because it is the key agent in contemporary colon cancer treatments [27]. Differences between cisplatin and oxaliplatin for their effects in colon cancer that are not well defined at the mechanistic level [28]. We plan to use *in vivo* models from our studies to investigate this in detail for Fap1 regulated events.

Although CRC-CSCs are CD133^+^, additional markers may be associated with functional activities of interest. Future studies in the laboratory will clarify if additional subpopulations of Fap1-overexpressing CSCs are specifically involved in relapse after oxaliplatin treatment or in metastasis.

Fap1-substrates of unknown significance for colon cancer pathogenesis include Iκbα (an inhibitor of Nfκb) and PDZ-Rho-GEF (a Rho activator) [29, 30]. These proteins will be the topic of future studies. It is also possible SLV peptide influences other proteins with PDZ-type protein interaction domains. Candidates would include Taz (involved in mesenchymal differentiation) or Dishevelled 1 (Dvl1; modular of Wnt activity) [31, 32]. These proteins will also be investigated in future studies. However, our current studies identified increased phosphorylation of Fas and Gsk3β in xenograft tumors from SLV peptide treated mice compared to mice treated with control peptide. This would not be anticipated with inhibition of Dvl1 or Taz.

We found Irf2 repressed *PTPN13* transcription in SW620 colon cancer cells. Inflammatory mediators, including Ifnγ and Tnfα, influence Irf2 expression and activity in a variety of cell types, resulting in termination of the inflammatory response [33]. In future studies, we plan to investigate the impact of inflammatory mediators on Fap1 expression and Fas sensitivity in CD133^+^ versus CD133^−^ colon cancer cells.

Our studies suggest that Fap1-inhibition after oxaliplatin chemotherapy might be a rational approach to colon cancer. Maintenance therapy is unusual in solid tumors, but is has an increasing role with current immunotherapeutic approaches [27]. Similarly, we previously found that the addition of SLV peptide to tyrosine kinase inhibitor (TKI) prevented emergence of TKI resistance or blast crisis in a murine CML model; events observed with TKI alone [20].

These studies suggest Fap1 may be a tissue agnostic target for malignant stem cells in diseases as diverse as colon cancer and CML. Normal stem cells that reside in colon crypts or the bone marrow niche balance self-renewal with differentiation to replace mature cells lost to programmed cell death on an ongoing basis. This process may require common regulatory mechanisms that are susceptible to derangement during transformation in both malignancies. Fap1 is also increased in head/neck cancer and glioblastoma, and these diseases may be of additional interest to better understand the role of Fap1 in stem cell biology and therapeutic targeting.

## MATERIALS AND METHODS

### Plasmids

*PTPN13* promoter sequences were amplified from the U937 cell line and reporter plasmids generated in pGL3-basic or pGL3-promoter vectors (Promega, Madison, WI), as described [21]. The cDNA for human Irf2 cDNA was obtained from Dr. Gary S. Stein (University of Massachusetts Medical School, Worcester, MA) and for Icsbp from Dr. Ben Zion-Levi (Technion, Haifa, Israel). Expression plasmids for Icsbp or Irf2, or shRNAs specific to Icsbp or Irf2 (or scrambled control shRNA) were previously described [21, 34].

### Cell lines

Colon cancer cell lines SW480 and SW620 were obtained from the American Type Culture Collection (ATCC, Manassas, VA) [24]. Cells were cultured in DME, 10% fetal bovine serum and 1% penicillin/streptomycin solution and authenticated annually by STR analysis according to manufacturer's instructions (GenePrint 10 system; Promega, Madison, WI).

### Quantitative real time PCR

Cells were lysed in Trazol for RNA isolation by standard techniques [21]. Oligonucleotide primers were synthesized by MWG Biotech (Piedmont, NC). Real time PCR was performed on an Applied Biosystems machine by the SYBR green/standard curve method. Six independent samples were assayed in triplicate for each experiment and results were normalized to 18S. A normal distribution was found for biological replicates.

### Reporter gene assays

SW620 cells (3 × 10^7^) were transfected by electroporation with reporter constructs (with firefly luciferase reporter, 20 μg) containing the *PTPN13* promoter (in pGL3-basic) or *PTPN13* cis element (in pGL3-promoter) with an internal control for transfection efficiency (TK-renilla luciferase, 2 μg). Some cells were transfected with vectors to overexpress or Icsbp or Irf2 (vs empty vector control; 5 μg of vector). Other cells were transfected with vectors to express shRNAs specific to Icsbp or Irf2 (or scrambled shRNA control vector; 5 μg). Luciferase activity was analyzed in triplicate for six independent experiments and normal distribution of values was observed for biological replicates.

### Flow cytometry

Adherent cells were detached with 0.05% Trypsin (Corning, Manassas, VA), washed and re-suspended in phosphate buffered saline (PBS). Tumors were mechanically and enzymatically disaggregated into single-cell suspensions, as in [35]. 1×10^6^ cells were incubated with PE-conjugated anti-CD133 and/or FITC-conjugated anti-CD44 (eBioscience, San Diego, CA; 12-1338-42 and 11-0441-82). Labeled cells were analyzed by LSR Fortessa Cell Analyzer (BD Bioscience). Data was analyzed with Flowjo software. At least three samples were analyzed in triplicate and a normal distribution was observed for biological replicates.

### Affinity purification of CD133^+^ cells

Cells were incubated with magnetic bead-conjugated anti-CD133 antibody and separated in a magnetic column, per manufacturer's instructions (Miltenyi Biotech, Auburn, CA; 130-097-049).

### Western blots

For Western blots of total lysate proteins, cells were lysed in RIPA buffer, and proteins were separated by SDS-PAGE and transferred to nitrocellulose. Membranes were serially probed with antibodies to Fap1 (Invitrogen, Carlsbad, CA; PA5-50660) and Tubulin (as a loading control). In other experiments, plasma membrane proteins were extracted (Abcam, Cambridge, MA; Ab65400) and Western blots performed, as described above. Membranes were serially probed with antibodies to Fas (Abcam; Ab110021) and Na^+^/K^+^ ATPase (as a loading control; Ab76020).

### Apoptosis assays

Some cells were labeled with anti-human FITC conjugated antibodies to CD133 (eBioscience, San Diego, CA), and others were separated with CD133 MicroBead Kit (Miltenyi Biotec, Auburn, CA). Apoptosis was assessed by flow cytometry using an Annexin V-APC Apoptosis Detection Kit according to manufacturer's instructions (eBioscience, San Diego, CA; 88-8007-74). In some experiments, apoptosis was induced with Fas-agonist antibody (monoclonal antibody clone CH-11, Millipore, Milwaukee, WI). Four independent experiments were performed in triplicate. Biological replicates were observed to have a normal distribution.

### Immunohistochemistry

Tumors were fixed in 4.0% paraformaldehyde, paraffin-embedded and sectioned. Slides were blocked after deparaffinization, rehydration and antigen retrieval; incubated with primary and secondary antibodies; and nuclei fluorescently stained with 4′,6-diamidino-2-phenylindole (DAPI) (Life Technologies, USA). Images were obtained with a Nikon A1R (20x). Antibodies include: anti-Fap1 or anti-Fas (Abcam, Cambridge, MA; ab198882, ab110021); anti-phospho Fas or anti-phospho Gsk3 beta (Thermo Fisher, Rockford, IL; PAS-38490, 702230); anti-Gsk3β (Novus, Littleton, CO; NBP1-47470); Cy3 AffiniPure Donkey Anti-Rabbit IgG, Cy2 AffiniPure Donkey Anti-Goat IgG, or Cy2 AffiniPure Donkey Anti-Mouse IgG (Jackson ImmunoResearch Laboratories, West Grove, PA; 711-165-152, 705-225-147, 715-225-150).

### Murine xenograft

Washed SW620 cells were re-suspended in a 50:50 mix of sterile PBS and matrigel (2.0×10^7^ per ml). Cell suspensions (0.1 ml) were injected in the right flank of athymic *Nude* mice (14-15 weeks old). Once tumors were 150-200 mm^3^, mice were randomized to receive SLV peptide or VLS control (5 mg/kg IP in 0.2 ml daily). Other mice were randomized to receive oxaliplatin (10 mg/kg) or saline control weekly for three weeks with concurrent SLV or VLS peptide. Investigators were not blinded in these studies. Animals were sacrificed when tumors were >2,000 mm^3^. Each cohort consisted of 12 animals to permit detection of a 15% difference between groups. There was a normal distribution of tumor growth within the cohorts.

### Statistical analysis

Data was analyzed by SigmaStat software. Differences of means were determined by two tailed Student's *T* test with standard error. One way ANOVA was used to compare more than two groups. Variance of groups within experiments was determined. Survival curves were compared by LogRank analysis. Statistical significance was p<0.02.

### Animal use approvals

Studies were approved by the Northwestern University Animal Care and Use Committee.
